# A snapshot of cancer in Chile II: an update on research, strategies and analytical frameworks for equity, innovation and national development

**DOI:** 10.1186/s40659-024-00574-2

**Published:** 2024-12-18

**Authors:** Cristóbal Vacarezza, Julieta Araneda, Pamela Gonzalez, Oscar Arteaga, Katherine Marcelain, Enrique A. Castellon, Ana Periera, Maroun Khoury, Bettina Müller, Juan Alberto Lecaros, Sofia P. Salas, Arnoldo Riquelme, Alejandro H. Corvalan, Jorge Jiménez de la Jara, Catterina Ferreccio, Carolina Goic, Bruno Nervi, Juan Carlos Roa, Gareth I. Owen

**Affiliations:** 1 Medical Public Health Residency Program, School of Public Health Dr. Salvador Allende, Faculty of Medicine, Universidad de Chile, Independencia 939, Santiago, Chile; 2 Servicio de Salud del Reloncavi, Esmeralda 269, Puerto Montt, Chile; 3 Public Health Program, School of Public Health Dr. Salvador Allende, Faculty of Medicine, Universidad de Chile, Santiago, Chile; 4 Faculty of Health and Odontology, Universidad Diego Portales, Santiago, Chile; 5 Faculty of Biological Sciences, Pontificia Universidad Católica de Chile, Alameda 340, 8331150 Santiago, Chile; 6 Millennium Institute on Immunology and Immunotherapy, 8331150 Santiago, Chile; 7 School of Public Health Dr. Salvador Allende, Faculty of Medicine, Universidad de Chile, Santiago, Chile; 8 Center for Cancer Prevention and Control (FONDAP-CECAN), Pontificia Universidad Católica de Chile and Universidad de Chile, Santiago, Chile; 9 Department Basic and Clinical Oncology, Faculty of Medicine, Universidad de Chile University of Chile, Santiago, Chile; 10 Institute of Nutrition and Food Technology (INTA), University of Chile, Santiago, Chile; 11 Laboratory of Nano-Regenerative Medicine, Faculty of Medicine, Universidad de Los Andes, Santiago, Chile; 12 IMPACT- Center of Interventional Medicine for Precision and Advanced Cellular Therapy, Universidad de Los Andes, Santiago, Chile; 13 Center for Bioethics, Faculty of Medicine, Clinica Alemana, Universidad del Desarrollo Santiago, Santiago, Chile; 14 Chilean Cooperative Group for Oncological Research (GOCCHI), Santiago, Chile; 15 National Cancer Institute, Santiago, Chile; 16 Bioethics and Law Observatory, Institute of Sciences and Innovation in Medicine, Universidad del Desarrollo, Santiago, Chile; 17 Department of Gastroenterology, Faculty of Medicine, Pontificia Universidad Católica de Chile, Santiago, Chile; 18 Department of Hematology and Oncology, Faculty of Medicine, Pontificia Universidad Católica de Chile, Santiago, Chile; 19 Advanced Center for Chronic Diseases (FONDAP-ACCDiS), Pontificia Universidad Católica de Chile, Santiago, Chile; 20 School of Public Health, Faculty of Medicine, Pontificia Universidad Católica de Chile, Santiago, Chile; 21 National Cancer Commission, Santiago, Chile; 22 Department of Pathology, Faculty of Medicine, Pontificia Universidad Católica de Chile, Santiago, Chile

**Keywords:** Chile, South-America, Research, Policy, Incidence, Clinical trials

## Abstract

**Introduction:**

Chile has achieved developed nation status and boasts a life expectancy of 81 + years; however, the healthcare and research systems are unprepared for the social and economic burden of cancer. One decade ago, the authors put forward a comprehensive analysis of cancer infrastructure, together with a series of suggestions on research orientated political policy.

**Objectives:**

Provide an update and comment on policy, infrastructure, gender equality, stakeholder participation and new challenges in national oncology. Assess the funding and distribution of cancer investigation. Present actions for the development of oncology research, innovation and patient care.

**Methods:**

Triangulating objective system metrics of economic, epidemiological, private and public sector resources together with policy analysis, we assessed cancer burden, infrastructure, and investigation. We analyzed governmental and private-sector cancer databases, complemented by interviews with cancer stakeholders.

**Results:**

Governmental policy and patient advocacy have led to the recognition of cancer burden, a cancer law, and a national cancer plan. Cancer has become the leading cause of death in Chile (59,876 cases and 31,440 cancer deaths in 2022), yet only 0.36% gross domestic product (GDP) is directed to research and development. Inequalities in treatment regimens persist. Prevention policy has lowered tobacco consumption, sugar intake via soft drinks and offered a high coverage of HPV vaccines. A high-quality cancer research community is expanding, and internationally sponsored clinical oncology trials are increasing.

**Conclusions:**

The cancer law has facilitated advancement in policy. Prevention policies have impacted tobacco and sugar intake, while gender equality and care inequality have entered the public forum. Cancer research is stagnated by the lack of investment. Implementation of a cancer registry and biobanking, reinforcement of prevention strategies, development of human resources, promotion of clinical trial infrastructure and investment in new technologies must be placed as a priority to permit advancements in innovation and equitable cancer care.

## Introduction

The objective of this “Snapshot” is to provide the national and international observer an insight into Chile’s current predicament, challenges and response to cancer. Approaching from the perspective of cancer investigation, this document will report on the cancer burden, risk factors, infrastructure and the public policies to address them, together with an analysis of basic and clinical research relating to financing, distribution and productivity within the country. To understand in more detail the clinical response of the Chilean health care system, a breakdown of national cancer epidemiology, and the future clinical challenges, we recommend the 2020 document “Addressing the rising burden of cancer in Chile: Challenges & opportunities” [1]. In the previous “Snapshot” publication we reported on the investigation-associated statistics in Chile between the years 2002–2012 and put forward recommendations for how the country could face the cancer burden. Herein, we present data on the decade 2013–2023 and comment on the progress of the previous recommendations, together with proposing new recommendations for the years ahead.

## Methodology

### Estimation of investment cost and distribution in cancer research

To estimate the investment in cancer research in basic and translational sciences, data from the projects awarded by the National Research and Development Agency (ANID) dependent on the Ministry of Science, Technology, Knowledge and Innovation, and from the Chilean economic development agency (CORFO) dependent on the Ministry of Economy, Development and Tourism were used. Information not available in publicly accessible databases was requested via the transparency law from the respective institutions (Request AH004T0005667 and AH004T0005826 on January 12, 2024 and January 28, 2024, respectively). Data on clinical trials was provided by the Chamber of Pharmaceutical Innovation (CIF) and by the Chilean Cooperative Oncological Research Group (GOCCHI). Additionally, information on financed research projects administrated by independent researchers or research groups related to oncology were obtained through interviews with the relevant stakeholders.

### Project inclusion criteria

Projects linked to oncology research executed between 2013 and 2023 were included, according to information contained in the title or summary of the project, or as reported by the informant. The variables of project type (basic and translational sciences or clinical trial), origin of the funding (national, international; private, public), instrument or program, sex of the researcher, responsible institution, region of execution and financing amounts were considered. When the project had more than one principal investigator, the total amount was subdivided according to the proportion of participation of each institution and responsible researcher, according to what was reported by the informants in personal interviews.

### Annual investment estimate

The annual investment estimate of financing was obtained from public databases or derived from the amounts reported by the investigators. When it was not possible to access the annual amount, the total value assigned prorated by the total time of the research project was used as a proxy for annual financing. Those projects reported in Chilean pesos or euros were transformed to the average value of the US dollar reported by the Central Bank for the corresponding year. In the case of the clinical trials reported by CIF, it was only possible to access the investment between the years 2018 and 2022, so the investment made between the years 2013 and 2017 was imputed using the average number of projects in 2018 as a reference together with the number of clinical trials carried out each year. The number and nature of clinical trials presented in this paper was requested through the Transparency Law from the Institute of Public Health (ISP) Chile (Request AO005T0008019, received on October 16, 2023). Similarly, the estimate of the investment corresponding to clinical trials in 2023 was made using the average financial value of the projects adjudicated in 2022 with the number of clinical trials registered that year in the ISP. In data management, StataSE 18^®^ and Microsoft Excel version 16.83^®^ software were used.

### Population projections

To estimate population dynamics according to their age composition, population projections from the Chilean 2017 CENSUS were used as the source [2]. Data management was carried out using StataSE 18^®^ software. The 2017 CENSUS was also used to report on the population numbers as this is the last official estimate that incorporates all national regions.

### Epidemiological data

Epidemiological data was obtained from the literature and all database references are included in detail in the reference section. World health and life expectancy data was obtained from the OECD Health Statistics database (https://www.oecd.org/en.html) [3] and the “Our world in data” website (https://ourworldindata.org/) [4]. Total population statistics on Chile were obtained from the World Bank and the International Monetary Fund (IMF) (https://www.imf.org/en/Home). Statistics searches were performed using national publicly available databases of ANID (https://anid.cl/), Ministry of Health (https://www.gob.cl/ministerios/ministerio-de-salud/), the Department of Health Statistics and Information (DEIS) (https://deis.minsal.cl/), the Chilean Institute of Public Health (ISP) (https://www.ispch.gob.cl/) with additional information from these organizations being requested through the transparency law. Cancer incidence and death were obtained from the International Agency for Research on Cancer (IARC) (https://www.iarc.who.int/), National Statistics Institute of Chile (INE) (https://www.ine.gob.cl/), World Health Organization (WHO) (https://www.who.int/), and Pan-American Health Organization (PAHO) (https://www.paho.org/en), databases.

### Estimation of the burden of disease from cancer

To estimate the burden of disease, publicly available data published by the Institute for Health Metrics and Evaluation at the University of Washington (https://www.healthdata.org/) an international initiative in which 160 countries and territories collaborate, was used together with data from Chile [5]. The proportion represented by the groups of diseases: neoplasms, cardiovascular diseases, musculoskeletal disorders, mental disorders and others, with respect to the total non-communicable diseases, in the Disability-Adjusted Life Years (DALYs), Years Lived with Disability (YLDs), Years of Life Lost (YLLs) and total deaths. The proportion of neoplastic diseases in DALYs, YLDs, YLLs and deaths was compared with respect to the other diseases for the years 1990, 2000, 2010 and 2019 and summary tables and figures were prepared. The document reports to 2019 to avoid distortions due to the COVID-19 pandemic. Data management was carried out using StataSE 18^®^ software.

### Interview process and data synthesis for health sector research

A bibliographic search was performed prior to interviews with stakeholders to prepare a thematic script and question list. Interviews with members of the health ministry and the private sector occurred between April and May 2024. At the end of the interview process, work meetings were held to synthetize the information and prepare the comments and recommendations for this manuscript.

## Results

### Demographics

**Fig. 1:**
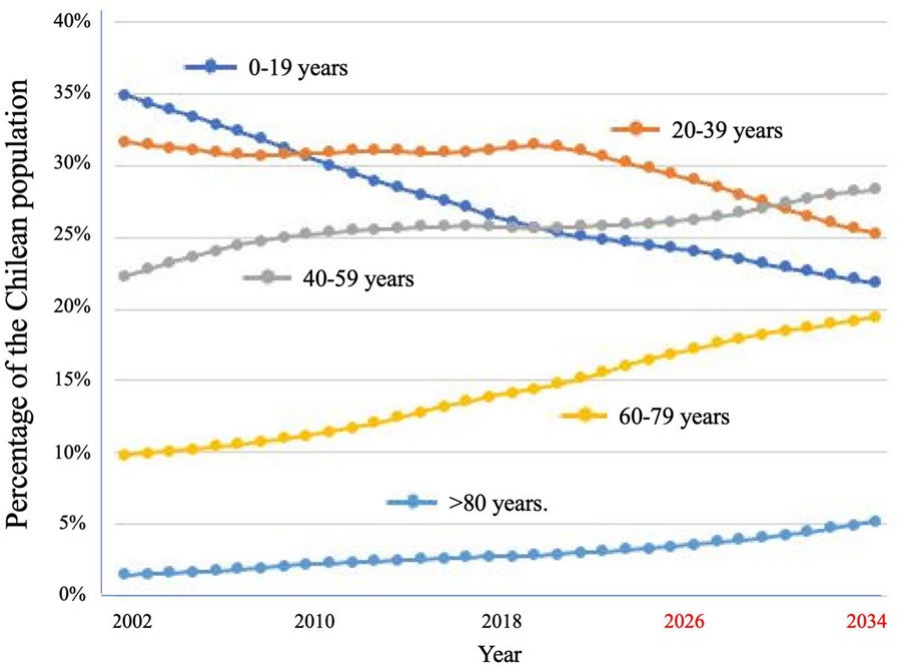
Aging of the Chilean population. Figures from 2002 to 2022 and extrapolations to 2034 (Data source of DEIS, Government of Chile)

In the last 40 years, Chile has experienced sustained economic growth, improvements in healthcare, a reduction in infant mortality to 5.6 per 1000 life births (DEIS, Chile) and the adoption of a Western lifestyle. In a population of now over 20 million, the current life expectancy in Chile is estimated to have returned to over 81 years after falling by 1.7 years during the COVID19 epidemic [6]. Chilean women currently have a life expectancy at birth of 83.3 years, among the highest in the world and overcome only by Canada (84.1 years) and Costa Rica (83.4 years) in the Americas [7]. This is also reflected by the increase in the aging population in Fig. 1. Chile is no exception to other countries that have adopted a western lifestyle. While live expectancy is increasing, birth rates are falling sharply and the percentage of people under 40 years of age is in decline (Fig. 1). This aging population brings with it an increased burden of cancer. If Chile is to witness a continued increase in life expectancy, efforts now need to focus on the prevention, early diagnosis and timely treatment of cancer. Furthermore, changes in Chilean immigration policy have led to the proportion of migrants increasing almost ninefold between 2006 and 2022, representing 1–8.8% of the country’s total population [8]. In 2021, 17.4% (30,829) live births that occurred in Chile corresponded to a mother of foreign origin [9]. As observed with the rise in the gallbladder cancer incidence in Sweden after the after large Chilean immigration in the 1970s, variations in incidence of certain cancer types in Chile will inevitably occur with the introduction of new genetic backgrounds [10].

The economy of Chile is a market economy and high-income economy as ranked by the World Bank. The nominal GDP per capita stood at $16,820 in 2023 [8, 9, 11] (having increased over threefold since the turn of the millennium, while the GDP at purchasing power parity was estimated at US$33,285 [12] In 2010 Chile became the first South American country to join the Organization for Economic Co-operation and Development (OECD) and is now considered one of South America's most prosperous nations. Directly related to the health of the nation and cancer statistics, between 2000 and 2022 poverty in Chile dropped from 36 to 6.5% (reported as poverty headcount ratio at national poverty lines as a percentage of the population) [13].

The public health insurer “Fondo Nacional de Salud (FONASA)” is funded through taxation and provides free or subsidized care to approximately 77.8% of the population in 2020. As health premiums are highly restrictive for low-income earners the private health insurance plans (ISAPRE) account for 17.2% of national coverage. The rest of the population corresponds to schemes related to armed forces personnel and individuals not affiliated with FONASA or ISAPREs [14]. Health spending in Chile stands at US$ 2699 per capita (2022) [2]. Most physicians work in the private health-care sector where greater financial incentives are incurred. Only 44% of physicians have contracts (often on a part-time basis) with public health providers. By OECD standards, the Chilean public sector is underfunded and ill-equipped, inevitably leading to inequality in access to specialist consultations, diagnostics, and surgery for cancer patients [15]. A clear inequity in the cancer survival rates in Chile as socio-economic condition worsens was recently highlighted in colorectal cancer. Five-year survival rates were 64% for ISAPRE (private) patients with only 31% for FONASA Group A (public) which covers the unemployed and immigrants [16]. The authors further reported that these discrepancies in patient survival time also correlated to geographical location and to the hospital where surgery/treatment was performed [16].

**Fig. 2:**
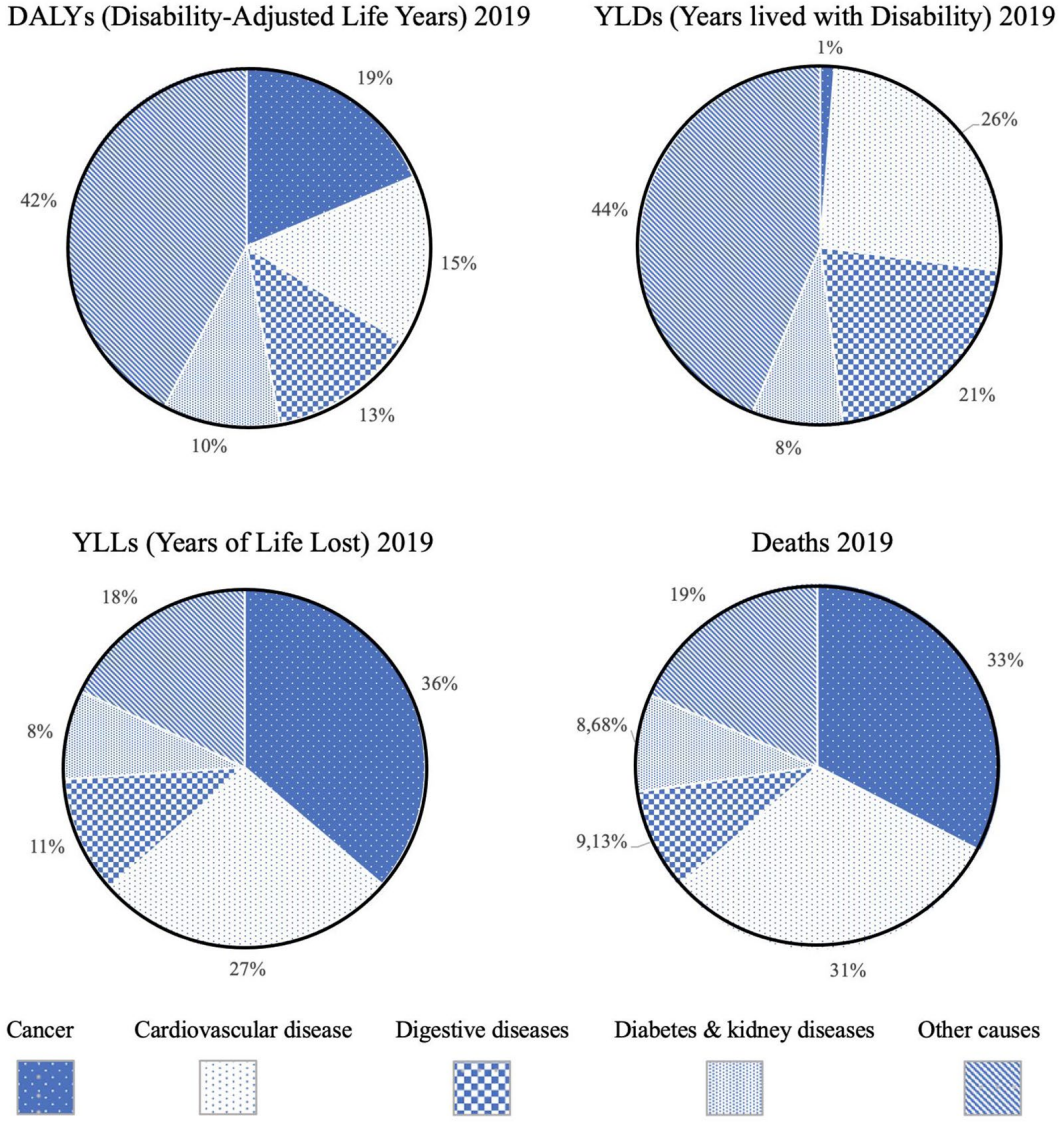
Chronic disease burden in Chile. *DALY* Disability Adjusted Life Years, *YLD* years of healthy life lost due to disability, *YLL* Years lost due to Death; DALY corresponds to the sum of YLD + YLL (Raw data source: Institute for Health Metrics and Evaluation & Chilean Ministry of Health 2019

As a country in development over the last century, Chile has focused its health concerns on combating communicable disease and creating a sustainable healthcare system. In1988 the National Program of Cancer Drugs for Adult (PANDA) and National Program of Cancer Drugs for Children (PINDA) were implemented. With the obvious exception of the COVID-19 pandemic, over the last decade Chile has taken steps to address cancer given the rise in incidence and negative economic indicators. Expressed as the number of years lost due to health problems, DALYs are a measure of overall disease burden. While not surprisingly cancer does not noticeable affect years of healthy life lost due to disability (YLDs), 19% of DALYs and 36% of potential years of life lost (YLLs) in Chile are due to complications and mortality from cancer (Fig. 2).

### Cancer incidence

Improved scientific understanding, sanitation, modern medicine, and social systems have allowed the average life expectancy at birth to increase from 32 years in the year 1900 to a current global average over 70 years [17]. Increased life expectancy and global control of communicable diseases have led to the positioning of cancer as the second cause of global mortality. The Global Cancer Observatory (GLOBOCAN) at the International Agency for Research on Cancer (IARC) estimated 19,965,054 cases worldwide in 2022 with 9,736,520 deaths. The Latin American and Caribbean region accounts for 7.8% of cancer incidence corresponding to 1,541,060 deaths in 2022 [18].

**Fig. 3:**
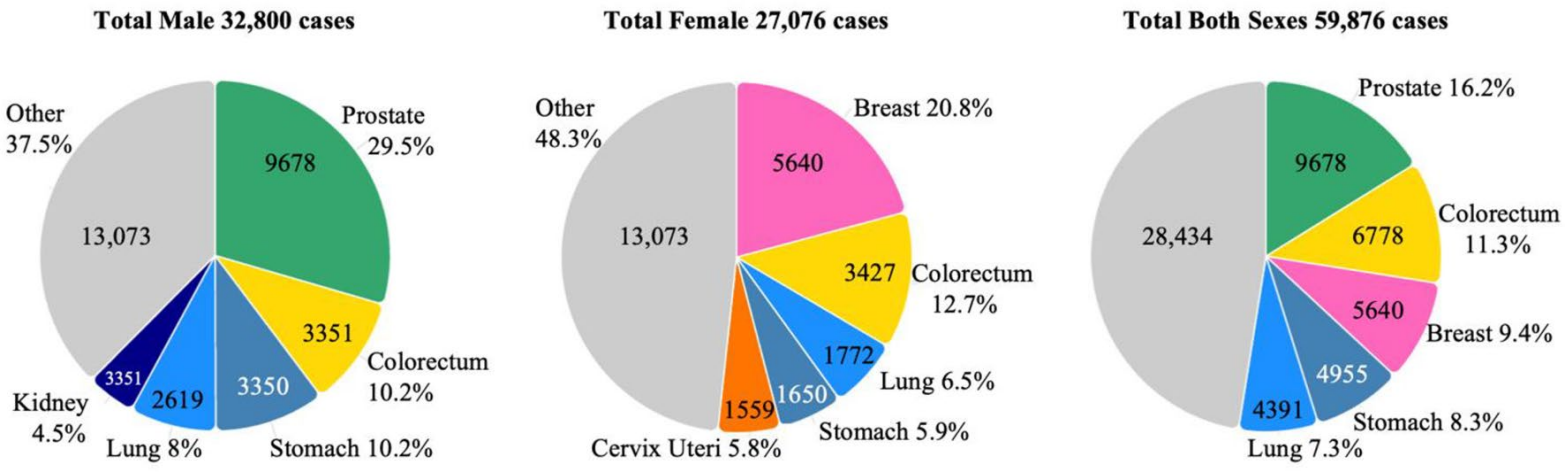
Cancer incidence in Chile by sex and tumor site (Raw data source: GLOBOCAN) [20]

Given that longevity is a leading indicator of cancer incidence, in the year 2019 malignant neoplasms became the leading cause of mortality in Chile after surpassing cardiovascular disease [19] (Fig. 2). In the year 2022 GLOBOCAN estimated an incidence of 59,876 cancer cases and 31,440 deaths in Chile [20]. The leading cancers (incidence ranked by cases) in men were prostate, colorectum and stomach, while in women the predominant malignancies were breast, colorectum and lung (Fig. 3). The “Integrated Cancer Control Initiative in Latin America (ICCI-LA) study”, published in 2020, estimated that in Chile cancer incidence will continue rising with a projected 74,973 new cases in 2030 and 94,807 new cases in 2040 [5]. Alarmingly, but in line with Chile’s aging population (Fig. 1), these estimated new cases represent a 38.3% increase between 2020 and 2030, and a 74.9% increase between 2020 and 2040 [5].

Several decades ago, Chile was notable for its high incidence of stomach, gallbladder and lung cancer [21].While breast, prostate and lung cancer mortality are slowly in ascendancy, encouraging news has been a stabilization in deaths from stomach, esophageal and testicular cancer together with lymphomas and leukemias. As with many countries in transition to a developed nation, together with the stabilization of stomach cancer, there has been a notable decline in gallbladder and cervical cancer mortality [22]. The reasons for the stabilization and not notable decline of stomach cancer are idiopathic, but may be related to chronic *Helicobacter pylori* infection, diet choices, and an increasing level of obesity in the country (discussed below).

The marked decline in gallbladder cancer mortality, which for years in Chile had one of the highest incidence rates in the world, may be attributed to a synergistic combination of factors, such as the significant improvement in hygiene conditions among the Chilean population, which has had an impact on the prevalence and incidence of salmonellosis and other bacterial infections. Additionally, better conditions for perishable food storage reduce the possibility of contamination with Aflatoxin, traditionally described as a risk factor for gallbladder cancer development. This is further compounded by the implementation of the governmental plan initiated by the Ministry of Health (AUGE-GES) in late 2007, aimed at conducting preventive cholecystectomies in patients aged between 35 and 49 with symptomatic cholelithiasis, a primary risk factor for gallbladder cancer. Since its inception, approximately 700,000 cholecystectomies have been performed, resulting in certain geographical areas where the proportion of cholecystectomized women in the at-risk age group exceeds 25% (women of indigenous origin in Chile are at a higher risk for gallbladder cancer).

Chile reflects the multitude of sociodemographic changes that have also occurred both in the world and in the South American region over the last decades, including urbanization and the progressive adoption of a more westernized lifestyle. As seen elsewhere, the mortality trends of infectious-related cancers have tended to decline, while rates of cancer types linked to westernization were mainly increasing. The changes in age stratification towards an aging population have brought expected increases in breast and prostate cancers (Fig. 3) [23]. In the case of breast cancer (Fig. 3), this increased incidence most is also likely coupled to a westernized trend in declining family size and lactation frequency. For example, births in Chile have fallen from 4.7 in 1960 to 1.54 per women in 2021 [24]. The last decades in Chile have witnessed a substantial rise in colorectal, pancreatic, kidney and liver cancer, offering new challenges to the healthcare system [25]. The incidence in colon cancer is strongly associated with the consumption of red meat, alcohol and processed foods, all factors classified as westernized diet [26]. Ultra-processed foods now represented 28.6% of the total energy intake of the Chilean population [27].

### Health risk factors in the Chilean population

The Global Cancer Observatory at IACR estimates that 169,882 cancer deaths could be prevented in Chile over a 5-year period [28] by lifestyle changes such as eliminating tobacco, reducing obesity, reducing alcohol consumption, promoting a healthier diet and by increasing physical activity. Moreover, cancer death could be greatly reduced by increased screening, vaccination, and early access to treatment. In Chile, tobacco use and high body mass index (BMI) were identified by national investigators to be the leading causes of preventable cancer cases [29]. They reported that approximately 30% of cancer cases (15,097 out of 50,320 cases) and 36% of cancer deaths (10,155 out of 28,010 deaths) in 2018 related to lifestyle risk factors.

As with any country having undergone a recent transition from underdeveloped to developed status, the malnourishment that was still present among poorer inhabitants up to the 1980’s has now been replaced by escalating obesity [30]. The most recent National Health Survey (2017) reported that 74.2% of Chilean adults have excess weight (39.8% are overweight, 31.2% are obese, and 3.2% suffer from morbid obesity), a figure that increased considerably from the 64.2% reported in the previous survey in 2010. An important cause behind these figures is likely to be sedentary lifestyle, as 86.7% of Chileans reported a lack of physical activity. The projected obesity in adolescent boys for the year 2019 was 19.25% and girls 13.97% [31]. In terms of economic burden, the medical costs of obesity reached $800 million, or 2.4% of all health care spending in 2016, a value predicted to reach almost 4% in 2030. In 2018 it was estimated that elevated BMI was attributable for 4394 cancer cases and 2572 and deaths [29].

A further alarming risk factor to national health and potential future cancer incidence is that now one in four pregnant women are obese [32]. Gestational obesity is associated with maternal metabolic alterations and more concerning with infant health problems that can be manifested throughout a lifetime [33–35]. Chile now needs to implement intervention strategies for a reduction in national BMI and especially consider pre-pregnancy obesity as a modifiable risk factor for the future health of the nation.

According to the National Food Consumption Survey, Chile falls short of meeting recommended dietary guidelines regarding water, fruit and vegetable, fish, legume, and dairy intake [36]. Notably, Chile ranks among the top countries in Latin America and the Caribbean (LAC) for per capita sales of sugar-sweetened beverages (SSBs) and exhibits elevated daily consumption of junk food, characterized by high levels of sugar, saturated fats, and sodium [37]. While Chile boasts comparatively high whole grain consumption, a significant portion is derived from refined white flour products [37]. Approximately 30% of total dietary energy in Chile is sourced from ultra-processed foods (UPFs), with carbonated soft drinks, cakes, cookies, sauces, dressings, gravies, reconstituted meats, milk-based drinks, fruit drinks or sweetened waters, and salty snacks being the most prevalent UPFs [38]. Demographic factors such as youth, metropolitan living, and higher income are associated with increased UPF consumption [37]. Moreover, local sales data indicates a substantial 59.8% increase in UPF and beverage sales between 2000 and 2013, suggesting a potentially escalating trend in UPF consumption in recent years [18].

## Legislatioin and public health policies in Chile

### Ministries of science and health

In 2018, Chile created “The Ministry of Science, Technology, Knowledge and Innovation” as a replacement for the “National Commission for Scientific and Technological Research” previously located in the Ministry of Education. This new Ministry oversees scientific research, technology transfer, public understanding of science, and technological-based companies in all regions of the country. However, the structuring, coordinating, and promoting of national science and technology is accompanied by a meager ministerial budget. The economic investment in science and technology is only 0.36% of Chile’s GDP in one the lowest of countries (OECD average is 2.74%) and out of line with other national economic indicators (e.g. GDP, GDP per capita).

The Ministry of Health has also been advancing in the last decade in innovations relating to oncology. In response to governmental recognition that immediate action needed to be implemented to address the increasing burden of cancer, “The National Cancer 2018–2028” was launched [22]. This action plan tackles health promotion and prevention, early detection, timely diagnosis of the disease, adequate treatment, palliative care, monitoring, and rehabilitation, with the objective of guaranteeing access to cancer care for the entire population. This National Cancer Plan was developed hand in hand with the implantation of a “cancer law” [39].

Patient advocacy in Chile in the field of cancer delivered benefits in 2012, when public pressure had a role to play in obtaining the biological treatment Herceptin and its extended use for breast cancer patients into the public health insurer (FONASA). The following year more than 10,000 people participated in the so-called “March of the Sick,” eventually giving rise to the “Ricarte Soto law” (Ley N° 20.850) in 2015 that contemplated the creation of a national solidarity and universal medicine fund, where the State contributed to support access to high-cost treatments [40]. After these successes, many of the patient advocacy groups related to cancer merged to have one voice in the association Asociación Chilena de Agrupaciones Oncológicas (ACHAGO) which worked together with the newly formed “National Cancer Forum” to lobby for a cancer law to guarantee a commitment from the government to oncology. The following years saw the incorporation of Senators of the Republic into the campaign, a draft law entered Senate, followed by numerous public events and marches of cancer patients within the country, to finally enact Law 21,258 “A National Cancer Law, *which pays posthumous tribute to Dr Claudio Mora*”. The cancer law was announced in 2020 and a National Cancer Commission was established. In 2022, the first working meeting was held between the Minister of Health and the National Cancer Commission to reinforce collaborative work with the civil society.

### The diet of a nation

Chile grapples with an escalating obesity epidemic, compounded by widespread physical inactivity and suboptimal dietary patterns. Addressing these multifaceted challenges through the implementation of robust health policies holds significant promise in mitigating cancer incidence rates.

### Food labeling law

The food labeling law and advertising began to be implemented in 2016 and completed its full implementation in 2019 (Law 20.606) (Table 1). This law involves front of package labels (FOPL) which are octagonal symbols in black and white that warn of foods high in sodium, sugars, total fats, and calories. Additionally, this law restricts the sale of “high in” products in educational institutions and limits targeted marketing of these products to children under 14 years of age [41]. Although the law has been implemented recently, benefits have already been observed. Families, adolescents, and children have increased their awareness of the seals and began using them to make purchasing decisions [42, 43]. This was also reflected in household food purchases, where there was a 24% reduction in sugary drinks and in purchases of “high in” foods (24% reduction in calories, 37% in sodium, and 27% in total sugars). It was also observed that the sugars and sodium content in foods available for sale decreased; the percentage of “high in sugars” products decreased from 80 to 60% (in beverages, milks and milk-based drinks, breakfast cereals, sweet baked products, and sweet and savory spreads), and “high in sodium” products decreased from 74 to 27% (in savory spreads, cheeses, ready-to-eat meals, soups, and sausages) [44]. Regarding marketing directed at children, improvements have also been observed; for example, advertising of healthy foods on broadcast television targeted at children and adolescents decreased from 50 to 13% [42]. However, there are still challenges in regulating and improving marketing on social media and the internet. Finally, improvements have also been observed in the quality of the diet provided in schools, although many adolescents report consuming more unhealthy products outside of school hours [45].

**Table 1:** Recent legislation in Chile affecting cancer control; Source Library of the National Congress Chile (BCN)

Year	Law/legislation	Impact
1995	Law 19,419 amended several times	Tabacco regulation
2013	Law 20,660	Health warnings at points of sale. Advertising is prohibited. The parent company / importer of tobacco products must annually inform the Ministry of Health on constituents and additives. No smoking in any closed public space or open space in establishments of education. Special designated areas to be provided in patios or open spaces
2022	Law 21,413	Environmental/sanatoria measures to prevent contamination from cigarette butts
1984	Law 18,290 amended several times	Alcohol regulation, drink driving
2012	Law 20,580	“Zero Tolerance law”. Accepted level of alcohol in the blood w reduced from 0.5 to 0.3 g per thousand of alcohol for drivers who drive under the influence of alcohol, and from 1.0 to 0.8 for those who drive while intoxicated
2021	Law 21,363	Further restrictions on commercialization and publicity
2014	Law 20,780 Tax reform	Taxing sugar-sweetened beverages Increased tax on produce from 14 to 18% if sugar content > 6.25 g /100 ml
2019	Law 20,606	Food Labeling Etiquettes on food packaging warnings of high sodium, sugar, total fats, and calories
2014 2021	Law 21,382 Amendment to Article 66a, which was an. amendment to Labor Code	Workers granted half a working day to receive preventative tests (mammograms, prostate exams, and cervical smears)
2015	Regulation ISP No. B-2554/15	HPV vaccine Free for adolescents
2015	Law 20,850 “Ricarte Soto law”	Creation of a national solidarity and universal medicine fund
2020	Law 21,258 A National Cancer Law, which pays posthumous tribute to Dr. Claudio Mora	Establish a regulatory framework that allows developing policies, plans and programs related to cancer, addressing all stages of disease management. Protection of the objectives of the Chilean National Cancer Plan, including a national cancer register

Details of each law can be obtained by entering the number of the law in the BNC website: https://www.bcn.cl/leychile/

### Sugar sweetened beverage tax

In 2014, Chile implemented a mixed methodology for taxing SSBs (Table 1). The previously existing tax for beverages was increased from 14 to 18% for beverages containing high levels of sugars (more than 6.25 g sugars/100 ml) and reduced from 13 to 10% for those beverages with lower sugars content. This tax policy did not affect plain water, dairy products, or 100% fruit juices. A recent study concluded that the policy effectively increased prices and reduced affordability [46]. Prices for SSBs with high sugars content increased by 2% for carbonated beverages and 3.9% for non-carbonated beverages. Conversely, prices for SSBs with low sugars content increased in some categories (ready-to-drink beverages by 1.5%) while decreasing in others (concentrates by 6.7%). Furthermore, household monthly per capita purchases decreased for SSBs high in sugar and increased for SSBs low in sugar [47]. While these results are encouraging for future policy making efforts, it may be premature to determine the impact of these regulations on national obesity rates.

### Tobacco control

Tobacco consumption has declined worldwide, with the percentage of people smoking declining from 22.7 to 17.7% between 2007 and 2021 respectively [48]. The latest “Report on Tobacco Control in the Region of the Americas 2022”, ranks the Americas as having the second lowest prevalence of tobacco consumption globally and now South America is categorized as smoke-free in public places [49]. This Region witnessed a reduction in the prevalence of current tobacco consumption from 28% in the year 2000 to 16.3% in 2020. However, the Pan American Health Organization (PAHO), signaled out Chile as the country with the highest prevalence of tobacco use within the American continent, reporting the adult prevalence of smoking at 29.2%, while the regional average is 16.3%. The Chilean Ministry of Health estimated in 2022 that 16% of the deaths in the country can be attributed to smoking. Annually, tobacco use is related to more than 62,000 cases of lung disease, 31,000 cases of heart disease, 12,000 Cerebral Vascular Accidents and more than 8500 presentations of cancer [50]. South American tobacco use is dominated by men and this pattern was historically the same in Chile. However, the feminization of tobacco consumption now sees 26.3% of adult Chilean women smoking, almost on par with men 31%. Disturbingly, at the time of the last survey in Santiago’s Metropolitan region, 20.3% of under fifteen-year-olds were using tobacco products and the consumption by adolescent women (26.4%) is now greater than that of adolescent men (13.5%) [51]. As action from policymakers was required, Chile joined the WHO Framework Convention on Tobacco Control in 2005. A comprehensive tobacco control act (amending Act No 19.419 of 1995) on smoke-free environments entered into force in 2013. As a result, smoking is now prohibited in all enclosed spaces accessible to the public or for collective commercial use, including restaurants, pubs, bars and casinos, and all indoor public places and workplaces, and on public transport. Smoking is also prohibited in outdoor areas of primary and secondary educational institutions. Although not totally prohibited, most tobacco advertising and promotion is also heavily restricted. The Ministry of Health issued four pairs of pictorial health warnings to be placed on either the front or back of the cigarette package, while terms such as “light” and “low tar” are prohibited on packaging.

### Alcohol and illicit drugs

Alcohol consumption is a principal risk factor for the development of cancer and one of its most preventable factors. In Chile, alcoholic beverages are taxed at an *ad valorem* rate of 31.5% for distilled products (spirits and liquors) and 20.5% for beers, wines and sparkling wines, after an increase in 2014 from 27% for spirits/liquors and 15% for beer [52]**.** A risk factor for breast, throat, esophagus, liver, and colorectal cancers (among others), in 2022 the monthly prevalence of alcohol consumption in Chile was 41.2% [53]. Encouragingly, in comparison to 2020, there has been a reduction in alcohol consumption in both men (51.5–45.1%) and in women (37.2–33.3%). The OECD estimates a consumption of 7.1 L per capita in 2019, which is lower on the scale for OECD countries [54]. In a recent Ministry of Health survey targeted at only people over 60 years of age, 66.5% of participants reported consuming the same amount of alcohol during the COVID-19 pandemic, while 26.7% say they have reduced their consumption and only 6.8% acknowledged having increased consumption [55]. While alcohol purchase is only permitted to over 18 years of age, and the government has recently reinforced measures to request identification for every person purchasing alcohol, the consumption and especially underage consumption is difficult to control. On containers with greater than 0.5 degrees alcohol, Law. 21,363 now requires printed graphic warnings of harmful effects. Another method had been to introduce a Zero Tolerance Law (actualization of law 18,290) on drinking and driving, with the aim of changing the national attitude on alcohol consumption (Table 1).

Regarding illicit drugs, a 2023 governmental study revealed that marijuana consumption remains stable (10.9%) and lower than the national peak reached in 2016 (14.5%). Cocaine paste also remains stable (0.3%) however, cocaine use that had fallen to 0.5% during the COVID19 pandemic has now returned to preexisting levels (0.9%) [53]. No national studies are currently available assessing illicit drug use and cancer incidence.

### Protection against cancer associated bacteria and viruses

#### Human papillomavirus (HPV)

Cervical cancer is the fourth most common cancer in women globally with approximately 660,000 new cases and 350,000 deaths reported in 2022. In Chile, cervical cancer is the fifth most common cancer in women (Fig. 3) and the second cause of death in women of reproductive age (15–49 years) [25, 56]. In 2014 the Chilean Ministry of Health identified the HPV vaccine (since 2024 the vaccine Gardasil 9: HPV Types 6, 11, 16, 18, 31, 33, 45, 52, and 58) as a main primary preventive measure and implemented free immunization against HPV for female adolescents [57, 58]. Partially against the evidence of the non-cost-effectiveness of vaccinating adolescent boys (due to the excellent herd immunity demonstrated when high coverage of girls is achieved), in 2019 the Ministry of Health expanded the vaccination program to all adolescents [59]. They also included in the secondary prevention strategies the use of HPV testing as an alternative to cytology-based early detection. The implementation has been uneven along the country based on the high cost of the tests currently used in Chile. Thus, HPV testing availability has become a new source of inequality. The government must open the market of HPV tests to new providers to make it available in low-resource settings. Furthermore, the use of self-testing which has demonstrated evidence in Chile [60] has not been implemented as an alternative to solve access or ideological barriers for the gynecological exams. Economic conditions exist in Chile to achieve a substantial improvement in cervical cancer. National investigators have suggested the replacement of the Pap smear with the HPV test, potentially with screening every five years with the option of self-sampling, and the incorporation of triage based on HPV 16/18 typing or Pap smear [61]**.**

#### *Helicobacter pylori* (*H. pylori*)

Gastric cancer is diagnosed at late stages with high mortality in Latin-American countries including Chile [25] with higher impact in low- and middle-income countries [62]. Gastric carcinogenesis is a multifactorial and slowly progressive process that provides an opportunity to diagnose at early stages, thus improving prognosis. Unfortunately, there is no routine screening for gastric cancer in Chile. Randomized clinical trials suggest that anti- *H. pylori* treatment reduces both incidence and mortality from this cancer by approximately 40% [62]. In Chile, more than 50% of adults are infected by *H. pylori* and thus a main strategy for gastric cancer primary prevention is eradication of *H. pylori* infection, recommended by international consensus including Maastricht/Florence and the first edition of the WHO Cancer Codex for the Caribbean and Latin-American Countries (CELAC region) [63]. The efficacy of the different eradication schemes is variable and has progressively decreased due to the increase in antimicrobial resistance [64, 65]. In Chile, the most effective therapies are quadruple therapies (concomitant quadruple therapy (four antibiotics) or Bismuth based quadruple therapy) [66] and based on recent evidence and high prevalence of Clarithromycin resistance, public policies including universal coverage for *H. pylori* therapy, should be updated [67]. On the other hand, secondary prevention should be addressed based on esophagus-gastric-duodenoscopy (EGD), as population-based screening using EGD has substantially reduced gastric cancer mortality in high-risk East Asian countries [68]. However, the public health service covers EGD only for symptomatic patients [67] Statistics from Asian populations, which possess similarly high cases of gastric cancer to that of Chile, demonstrate that a significant proportion of cases and deaths can be avoided if preventative actions are adopted. Recently a strategic framework to deliver effective prevention and control of gastric cancer in the Americas was published. Table 2 summarizes the principal recommendations, rationale, and potential actions for prevention that could be implemented in Chile.

**Table 2:** Recommendations, rationale, and potential actions for gastric cancer prevention and control in Chile (and the Americas). Adapted from Riquelme et al. [69]

Recommendation	Rationale	Potential implementation actions
Strengthen population-based cancer registries	As the epidemiology of GC is changing in the Americas, periodical, and detailed registry-based analyses are required The Chilean Cancer Law now requires a national cancer registry. High-quality and comprehensive cancer registries will ensure that policymakers have accurate and timely data on incidence, treatment, and survivorship to make decisions based on evidence	Implement a sustainable registry with good national coverage and adequate funding Establish new population-based registries in high-risk areas with no coverage
Support development and dissemination of standards for quality care	High quality must be a requirement for health care providers in Chile	In collaboration with the Ministry of Health, clinical and academic societies promote multidisciplinary and establish combined operational guidelines and promote consistent reporting of histopathologic findings
Enable training of health care workforce	Trained health providers can effectively limit morbidity and mortality associated with GC	Establish high-level training for health providers in areas of *H. pylori* eradication and endoscopy practice and biopsy taking
Establish *H. Pylori* management registration and a surveillance system of antibiotic resistance	Promote quadruple clarithromycin-based therapies. Registry data of *H. pylori* for the selection of regimens to be used in each population based on studies of antibiotic resistance in the same population	Establish a large-scale long-term prospective registry of *H. pylori*-positive patients receiving eradication therapy
Perform endoscopic campaigns in high-risk populations (e.g. rural areas), focusing on individuals with major risk factors	Mobile screening services have served to expand access to cancer screening in diverse contexts as individuals in lower socioeconomic and indigenous groups exhibit higher risk of GC incidence and mortality	Perform endoscopic campaigns focused on high-risk individuals (i.e., adults aged ≥ 50 years, male sex, smokers, family history of GC) and ideally symptomatic
Strengthen strategies to reduce salt (sodium) intake	High salt intake increases the risk of GC by synergizing with the pathogenic effects of chronic *H. pylori* infection	Adopt WHO recommendations for salt intake reduction to reinforce salt reduction interventions in primary-care settings

#### Epstein–Barr virus (EBV)

EBV is a linear double-stranded DNA that belongs to the *Herpesviridae* family [70]. While best known as the cause of infectious mononucleosis in adolescence, EBV establishes a persistent infection in adulthood in more than 90% of the human population [71]. Initially described in lymphomas and nasopharyngeal carcinoma [72] persistent EBV infection currently represents a subtype of the molecular classification of gastric cancer [73]. An international pooled analysis reported a worldwide association from 1.9% in the Middle East to 16.8% in Latin America, particularly Chile, where a high prevalence has been reproduced [74, 75]. EBV-associated gastric cancer is characterized by the presence of a unique strain, whereas multiple strains can be found in healthy donors [76]. While eradication of EBV is not an alternative at this moment in time, it is interesting to note that the EBV-subtype of gastric cancer has a better prognosis and an exceptional response to immune checkpoint inhibitors [77], suggesting the implementation of targeted interception strategies for EBV-associated gastric cancer in Chile.

## Infrastucture for cancer policy and research

### Cancer registries and biobanking

Currently cancer incidence rates are obtained from epidemiological registration and surveillance systems, which presently incorporate seven population registers in the country, covering an estimated 21.8% of the population. Some of these systems are IARC registered and are used for global data collection. The majority of current data comes from hospital cancer registries and death certificate statistics.

Chile now acknowledges that the effective implementation and monitoring of interventions and cost-effectiveness in cancer care are contingent upon the existence of a comprehensive functioning registry. These registries must be designed to not only facilitate but also make transparent the registration data to researchers and the national scientific community. This goal could be achieved through the development of a digitalized national information system for oncological health information.

Historically in Chile, the reporting of both pediatric and adult cancer has been mandatory. Health institutions, especially pathology services, are obligated to periodically report patients diagnosed with cancer to the regional ministerial health secretariats (SEREMI). However, unlike pediatric cancer, which benefits from an updated and systematized registry, the adult cancer registry has faced budgetary deficits in both human resources and administrative infrastructure, hindering the enforceability of this obligation.

A national cancer registry with ready accessible information and an equally accessible biobank is a stand-alone research institution. Currently, there are only three tumor biobanks in Chile (Pontificia Universidad Católica de Chile, Fundación Arturo Lopez Perez and Universidad de Chile) that function effectively following harmonized protocols for recruitment, processing, storage, and sample sharing. The importance of having high-quality samples linked to relevant clinical information is acknowledged, despite this, neither the Ministry of Health or the Ministry of Science, Technology, Knowledge and Innovation have not generated specific Biobank financing initiatives, which makes the establishment of a network unfeasible. Repeated efforts to secure funding for the implementation of a national network of tumor banks have been unsuccessful unfortunately the Ministry of Health and the Ministry of Science, Technology, Knowledge and Innovation have not generated specific Biobank networks financing initiatives, which makes the establishment of a network unfeasible.

This lack of long-term perspective for an organized network of repositories goes against evidence generated in Spain, when in 2010 a national network of tumor banks was established [78]. This network has proven to have an impact on both the quantity and quality of new knowledge generated as well as on the economic aspects of the biotechnological industry in that nation [79]. Furthermore, the absence of clear and current regulations governing the operation of ethics committees in relation to biobanks in Chile has hindered the implementation of new nodes and the functioning of existing ones. The integration of a network of biobanks with a computerized national cancer registry, made available and connected with a digital medical record system that respects patient rights, will undoubtedly be a fundamental tool for the quality of national cancer research. An example to follow is the UK Biobank, which has already been recognized as a globally important resource for cancer research [80]**.** Currently over 26,000 researchers worldwide have assessed samples or data to perform cancer research. This initiative has already enabled research into the both the determinants of cancer and future planification of cancer policy and thus, could be used to lobby the Chilean ministries to promote biobanking within the country.

### Population cohorts to measure the health of Chile

Administrated by the Advanced Center for Chronic Disease (FONDAP- ACCDIS), Chile has established a population cohort (termed MAUCO) in the mid-southern region of Chile [81]. The cohort in the Province of Curicó seeks to analyze the natural history of chronic diseases in an agricultural community of 40,000 inhabitants. Its epidemiological interest stems from the drop in the poverty rate over the last few decades and from going from an undernourished population to possessing excess caloric intake. Furthermore, the area presented the highest national rates stomach cancer, gallbladder cancer and cardiovascular disease and the time of recruitment. This study has completed enrollment and had an initial follow-up of 10,000 adults aged 38–74 years. Enrolled individuals completed a survey exploring risk factors such as diet, alcohol, physical activity and pesticide exposure, together with an electrocardiogram, abdominal ultrasound and bio-impedance and hand-grip strength tests. Blood, urine, and saliva samples were taken [82]. The maintenance, further investment and potential expansion of this cohort will help design public health interventions tailored to agricultural populations in Latin America.

A further national cohort is at The Center of Innovation in Health (ANCORA) at the Pontificia Universidad Católica de Chile. This multidisciplinary team focuses on designing, implementing, evaluating and systematizing complex changes in the national healthcare system. The ANCORA cohort has been implemented in three hospitals and seven primary care centers a patient-centered complex intervention for adults with multimorbidity and proposes to implement solutions to gaps in the national health system [83].

## Basic and clinical cancer research in Chile

**Fig. 4:**
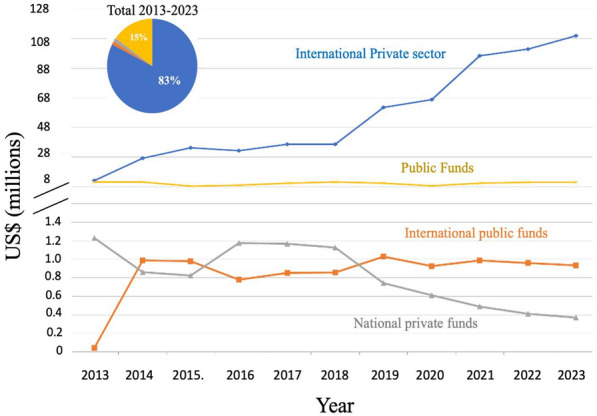
Investment (US$) in cancer research in Chile by funding source and year. The total funding of cancer research including international private sector (international funds for basic/translational research and sponsored clinical trials, national private and public funding and national private funding

The OECD has classified Chile, Colombia and Costa Rica has having the lowest investments in research and development (R&D) per GDPs and possessing the lowest number of investigators in R&D of the 36-member countries. Chile’s investment of only 0.36% of its GDP on R&D is hindering the development of it’s *know how based* economy. Concentrating solely on cancer research, the Chilean state invested less than US$11 million in 2023 on investigation for a disease that accounts for 33% the population’s mortality. Resources from the national or resident international private sector (e.g. *Center of Excellence in Precision Medicine -Pfizer Chile (CEMP))* is scarce, with no more than US$1 million invested per year. A similar sum of money enters the country as Chileans participate in international grants such as the US-NIH or the European Union Horizon 2020 (international public funds on Fig. 4). Encouraging, and discussed separately in a section below, the foreign investment in oncology in Chile, primary on clinical trials, is healthier, accounting for an estimated spending of over US$100 million in 2023 (Fig. 4).

**Fig. 5:**
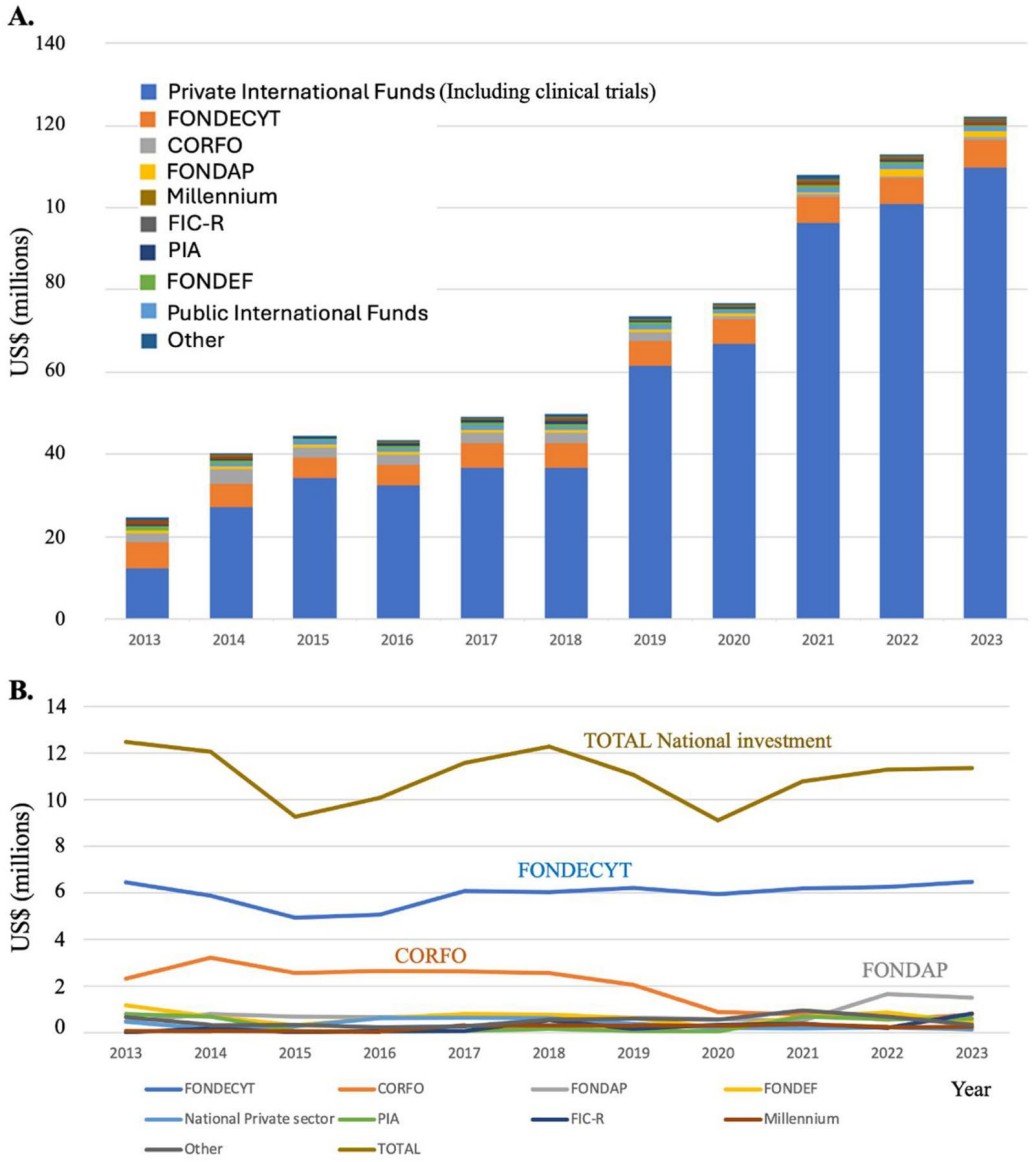
Investment in cancer research in Chile by funding agency. **A** The total funding of cancer research including international sponsored clinical trials, national private and public funding and international public funding. **B** Investment in cancer research in Chile from national governmental and the national private sector

Data from the previous version of this “Snaphot” paper [21] showed that in 2003 the total national investment in cancer research was approximately US$1 million and this rose steady to US$12 million in 2012, before declining and plateauing over the last decade (Fig. 4). Given the substantial increase in imported goods (reagents for scientific use) in the last decade, this plateau signals a real-world decline in the national scientific investment in cancer. The National Research and Development Agency (Agencia Nacional de Investigación y Desarrollo, ANID) located within the Ministry of Science, Technology and Knowledge awards investigator-led research FONDECYT projects (similar to RO1 grants in the USA) averaging US$72,0000 per year for 3–4 years (including institutional overheads), and also has a similar call for junior investigators (within a 10-year period since obtaining thew title or M.D or Ph.D. It is FONDECYT that maintains the foundation of national cancer research, funding on average US$ 6 million in grants per year) (Fig. 5). ANID also offers post-doctoral fellowships that cover principally salary. While these grants allow the development of good quality science, the numbers of grants being awarded has dropped from around 45% to below 39% in the last decade [84] However, given that laboratory-based science is more expensive than other disciplines, when the funding sections that principally fund cancer research (FONDECYT Regular, areas of Biology and Medicine) are accessed, only 31% of grants were approved in 2023 as opposed to 58% in 2013 [85, 86]. While this approval percentage may seem high by international grant standards, it needs to be taken into consideration that only one grant call exists per year in Chile and there is little alternative to obtain other funds for basic research. This scenario now leaves a high percentage of cancer investigators in the country without funding.

Further deepening the crisis, is that the State offered scholarships for doctoral studies outside of Chile with the condition that the student returns to Chile on completion of their studies. Unfortunately, this influx of doctors leads to an increase in funding applications with no extra university positions or FONDECYT resources being made available. Although lower in value to FONDECYT grants, FONIS grant funding exists for quality-of-care studies in clinical-related settings. CORFO investigation funds, now part of ANID, also offer funding for applied projects that have or may lead to patents (FONDEF). These projects are scarce and often require co-investment from the private sector. Further initiatives come in the form of centers of excellence such as Consortium, Millennium, Basal and FONDAP. The ANID Millennium Scientific Initiative finances and supports research centers of excellence of which 17 Institutes and 36 Nuclei exist [87]. One Institute, The Millennium Institute on Immunology and Immunotherapy invests resources in cancer research. The Financing Fund for Research Centers in Priority Areas (FONDAP) currently has two centers dedicated to cancer research, the previously mentioned ACCDIS and the Center for Cancer Prevention and Control (CECAN). The combination of these projects accounts for the increase in FONDAP funding from 2021 in Fig. 5. While these projects are well financed and productive, they are scarce and only reach a small proportion of the R&D community. The Associative Research Program (PIA, Basal) emerged in 2006 with the purpose of coordinating various instruments and initiatives to support associative research and the promotion of research centers of excellence. The Science & Life Foundation (Fundación Ciencia y Vida), the Center for Aging and Regeneration (CARE) and the Universidad de Los Andes received money from this program that in some part helped finance cancer research. The Innovation Fund for Regional Competitiveness (FlC-R) also has made funds available for cancer research (Fig. 5).

The authors predict that the effect of deprioritizing investigation in the last decade will have a long-term effect on Chilean science. The morale in scientific environments is low and many students are pursuing other careers or not entering higher education (e.g. entering doctoral programs) based on the presumption that there will be no funding for investigation or an academic future upon graduation.

### Distribution of cancer research funding in Chile

**Fig. 6:**
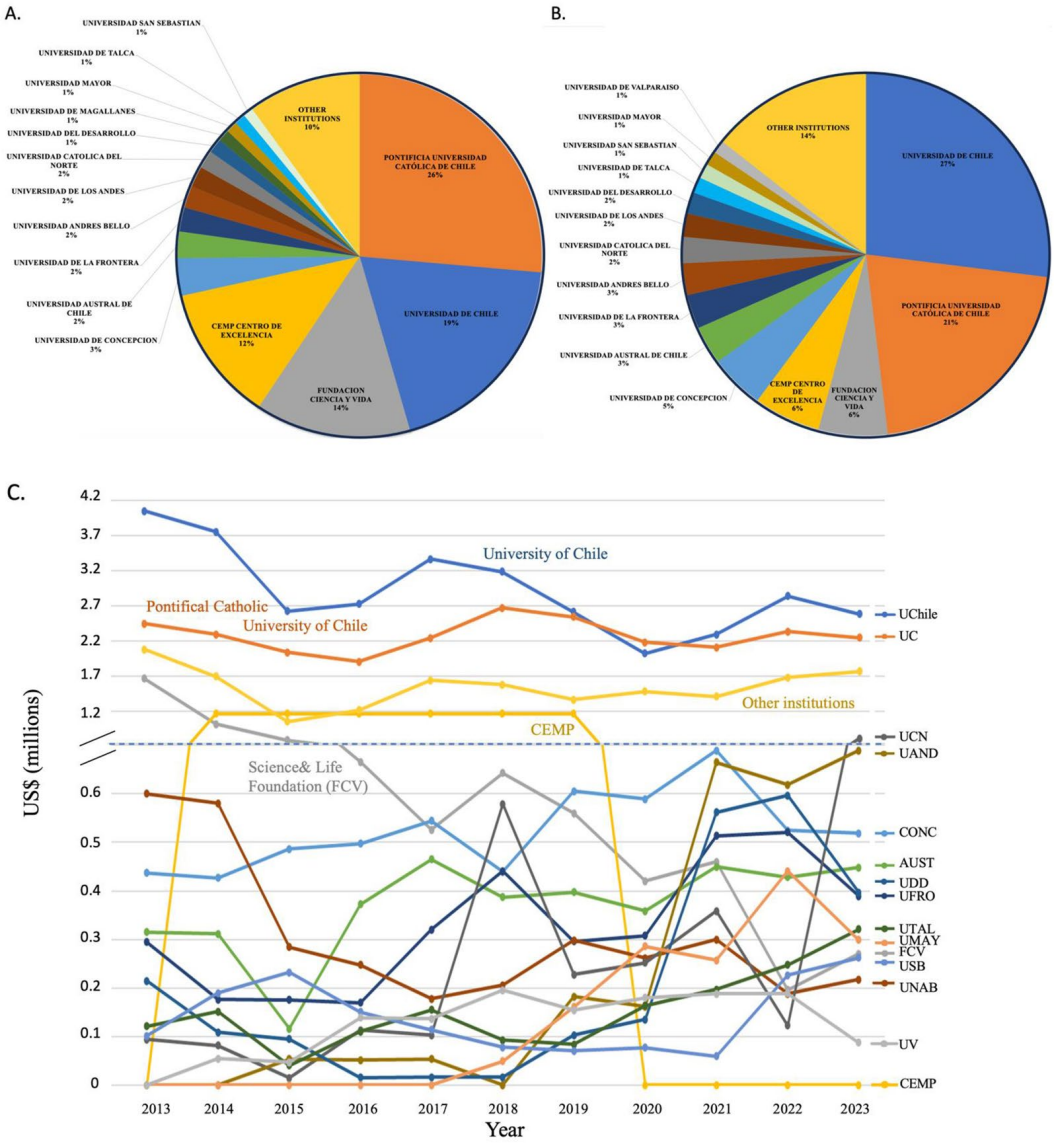
Investment in Cancer Research in Chile by host institution. **A** The total cancer research funding received by institution (not including clinical trials) from all funding agencies as percentage of total expenditure from 2013 to 2023. **B** The total cancer research funding received by institution (not including clinical trials) from only national funding agencies as percentage of total expenditure from 2013 to 2023. **C** The total cancer research national funding received by institution per year (not including international funds). Abbreviations: Universidad de Chile (U. Chile), Pontificia Universidad Católica de Chile (UC), Universidad Católica del Norte (UCN), Universidad de los Andes (UAND), Universidad de Concepción (CONC), Universidad Austral (AUST), Universidad de Desarrollo (UDD), Universidad de la Frontera (UFRO), Universidad de Talca (UTAL), Universidad Mayor (UMAY), Fundación Ciencia y Vida (FCV), Universidad San Sebastián (USS), Universidad National Andres Bello (UNAB), Universidad de Valparaiso (UV), Center of Excellence in Precision Medicine -Pfizer Chile (CEMP)

In the Chilean higher education system, there are 46 universities accredited by the Chilean National Accreditation Commission, representing 94% of the total student registration. For numerous years Chilean higher education and specifically the location of cancer research was dominated by the public “Universidad De Chile” (U. Chile) and the private universities the “Pontificia Universidad Católica de Chile” (UC) and the “Universidad de Concepcion”. While the former two universities undertake the highest proportion of Chilean research in cancer (among most areas), many new private universities have invested in this area. The percentage of funding received by each university or institution between 2013 and 2023 is shown in Fig. 6. Although the U. Chile and the UC have a majority share, in recent years the private institutions “Universidad del Desarrollo”, “Universidad San Sebastian”, “Universidad Nacional Andres Bello”, “Universidad Mayor” and the public institutions “Universidad de la Frontera” and “Universidad Católica del Norte” now have notable investigators and funding in cancer research.

The non-university institutions with notable funding on Fig. 6 are the Science & Life Foundation and the Center of Excellence for Precision Medicine (CEMP). Science & Life Foundation is a private science park that had an extremely promising cancer therapy which entered national and international clinical trials after years of basic research in Chile [88]. After obtaining a high level of funding for several years, their principal line of cancer investigation has now closed. CEMP was an initiative of CORFO to attract the pharmaceutical giant Pfizer to perform biomedical investigation in Chile. CORFO provided co-financing of US$7 million over a period of 5 to 6 years, which added to US$14 million contributed by Pfizer, making up a total investment of US$21 million. The CEMP research focused on the field of lung oncology, seeking to validate new technological platforms for molecular diagnosis based on next-generation genomic sequencing. Unfortunately, the Center received no further funding after the initial investment and announced closure in 2019.

**Fig. 7:**
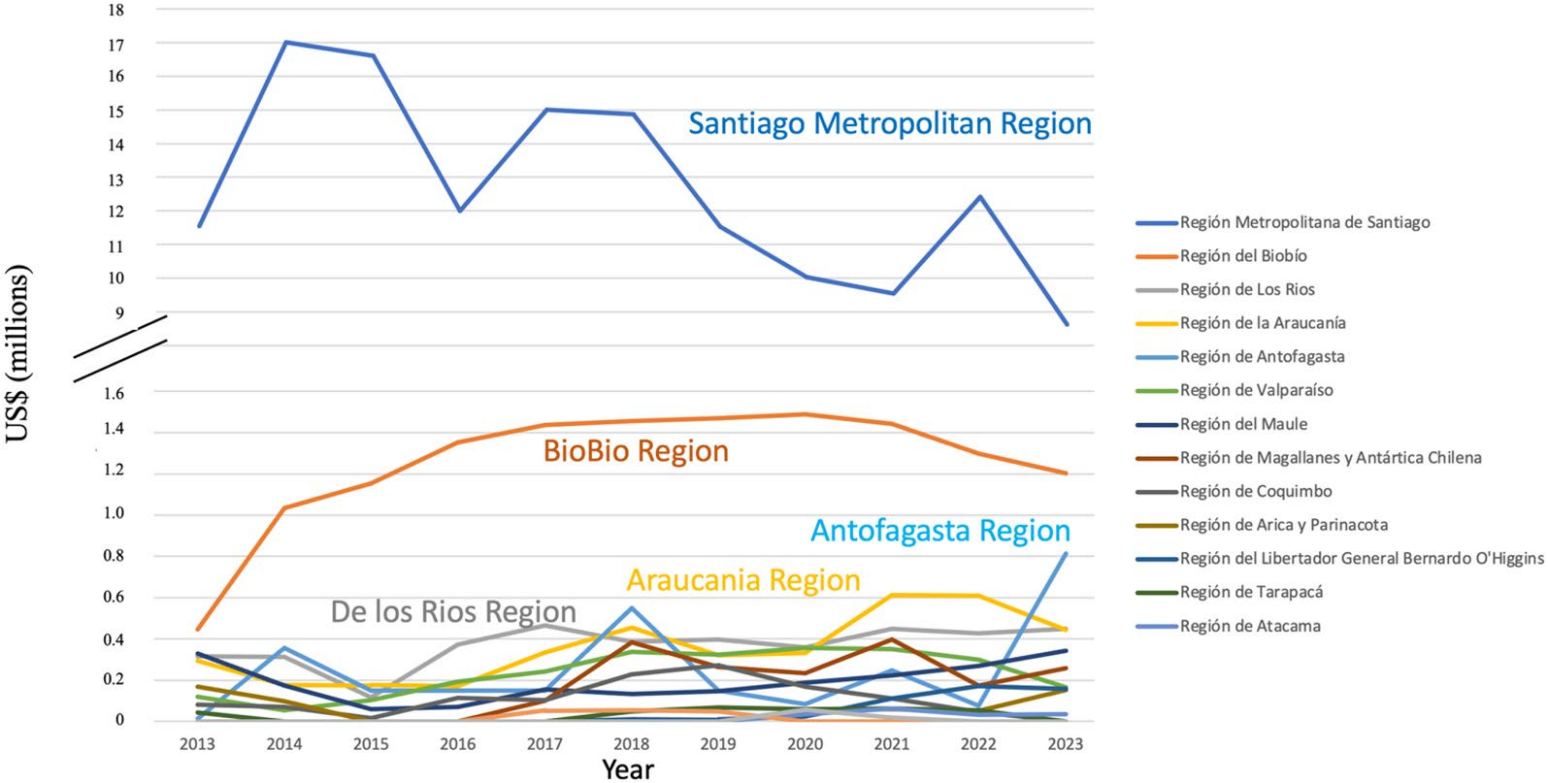
Investment in Cancer Research in Chile by Region. The total cancer research funding received by regional institutions from national and international funding agencies (not including clinical trials)

Given that the Santiago Metropolitan area accounts for approximately 40% of the national population and considering the number of projects obtained by the Santiago based U. Chile and UC, it comes as no surprise that this region that dominates the investment in cancer research in the country. However, Fig. 7 demonstrates that there is a tendency for decentralization with BioBio, Araucania, De los Ríos and Antofagasta regions maintaining and gaining national funding. Along with specialization and recruitment in the area, the regional competitiveness FlC-R grants have contributed to this increase.

### Clinical trials in oncology in Chile

**Fig. 8:**
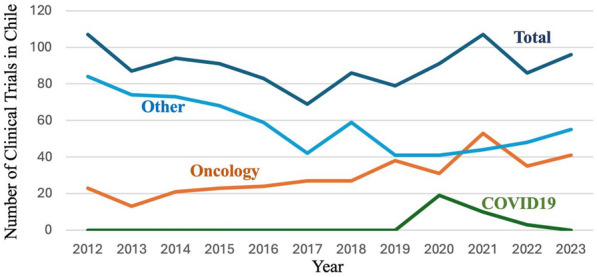
Clinical trials in Chile 2012–2023. Total clinical trials and clinical trials in oncology registered with the Chilean institute of public health between 2012 and 2023. COVID19 and other clinical trials and are shown as a reference. Source ISP Chile

In 2022, the distribution of Global Clinical Studies was concentrated primarily in North America (35.3%) and Europe (23.8%), however 2.4% of trials were performed in South America. [89]. While Brazil (47%), Argentina 25% and Chile (12.5%) host the highest number of clinical trials, Chile leads the region in number of trials per capita (4.6 clinical studies/million inhabitants). Of the clinical trials in Chile, between 20 and 50% are directly related to oncology each year (Fig. 8).

Due to a greater awareness of clinical trials and national websites (e.g. https://estudiosclinicos.cl/), cancer patients are increasingly seeking participation and, as a result, several private centers (for example Bradford Hill, James Lind and Saga) have been established in the country offering participation on clinical trials in oncology [90–92]**.** While the vast majority (99%) of clinical trials are international pharmaceutical industry phase III, principally operated by the Chamber of Pharmaceutical Innovation (Cámara de Innovación Farmacéutica CIF), there have also been several national investigator-led early phase trials in Chile, notably from the Science and Life Foundation and the Universidad de Chile [88]. Formed in 1997, the Cooperative Group for Oncological Investigation (Grupo Oncológico Cooperativo Chileno de Investigación, GOCCHI) is a non-profit organization which operates clinical trials in national public hospitals. Already a coordinator for international charities, such as the Breast International Group (BIG), GOCCHI is now working with the South-Western Oncology Group (SWOG) to conduct US-NIH funded trials in Chile [93].

**Fig. 9:**
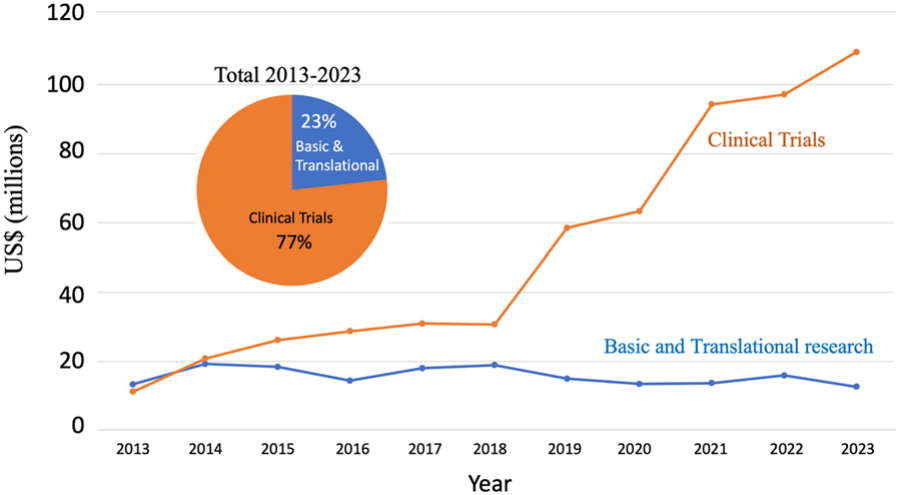
Investment in clinical trials and basic and translational research in Chile 2013–2023. All sources of Clinical trials and basic/translational research

The increase in clinical trials expenditure is multifactorial, while more clinical trials are being performed in Chile, the cost of these trials are not increasing proportionally. Between 2018 and 2022 the number of clinical trials in oncology increased from 27 to 35 M US$, however the investment increased more than three-fold (Figs. 8 and 9).

Chile, and South America in general, offer tremendous and still underutilized potential to perform clinical trials. Public and private health systems in the region only reimburse one or two lines of therapy, and thus patients undergoing trials in South America have not had accesses to the further lines of treatment available in North America or Europe. Furthermore, it is increasing evident that not all regions of the world have the same genetic make-up and thus it is a moral responsibility to test the efficiency of cancer treatments outside the North American and European populations. As with all countries, clinical research in Chile is subject to several health codes, namely: the Chilean Health Code; Law 19,628 on personal data; Law 19,779 that establishes special rules in relation to prevention, diagnosis, treatment and research in people with human immunodeficiency virus; Law 20.584 that regulates rights and duties linked to healthcare; Law 20,120 that regulates scientific research on human beings; Exempt Decree No. 32/2013 of the ISP on the Standard of Good Clinical Practice; Decree 62 that refers to the operation of the Ethics Committees and Law 20,850 on financial protection of high-cost diseases.

Of the 73 clinical trials conducted in oncology between 2012 and 2013, 63% were performed in the private sector. To increase the attraction of clinical trial sponsors to the country, the infrastructure and legislation in public health care system needs to be addressed. Initiatives such as the creation of a Department of Research, Development, Innovation and Dissemination at the Metropolitan Hospital of Santiago or the creation of the category of university hospital promoted by the Ministry of Health (Law 21.621) and its investigative function, should promote ethical responsibility of public institutions in the development of new and more relevant treatments for patients. This task is multifactorial and will need to include a Ministry driven initiative to demand enrollment, dedication of time of medical staff in hospitals, together with training and infrastructure. All patients, irrespective of socio-economic status, should have the option to participate in a clinical trial.

### Analysis of scientific productivity in Chilean cancer research

**Fig. 10:**
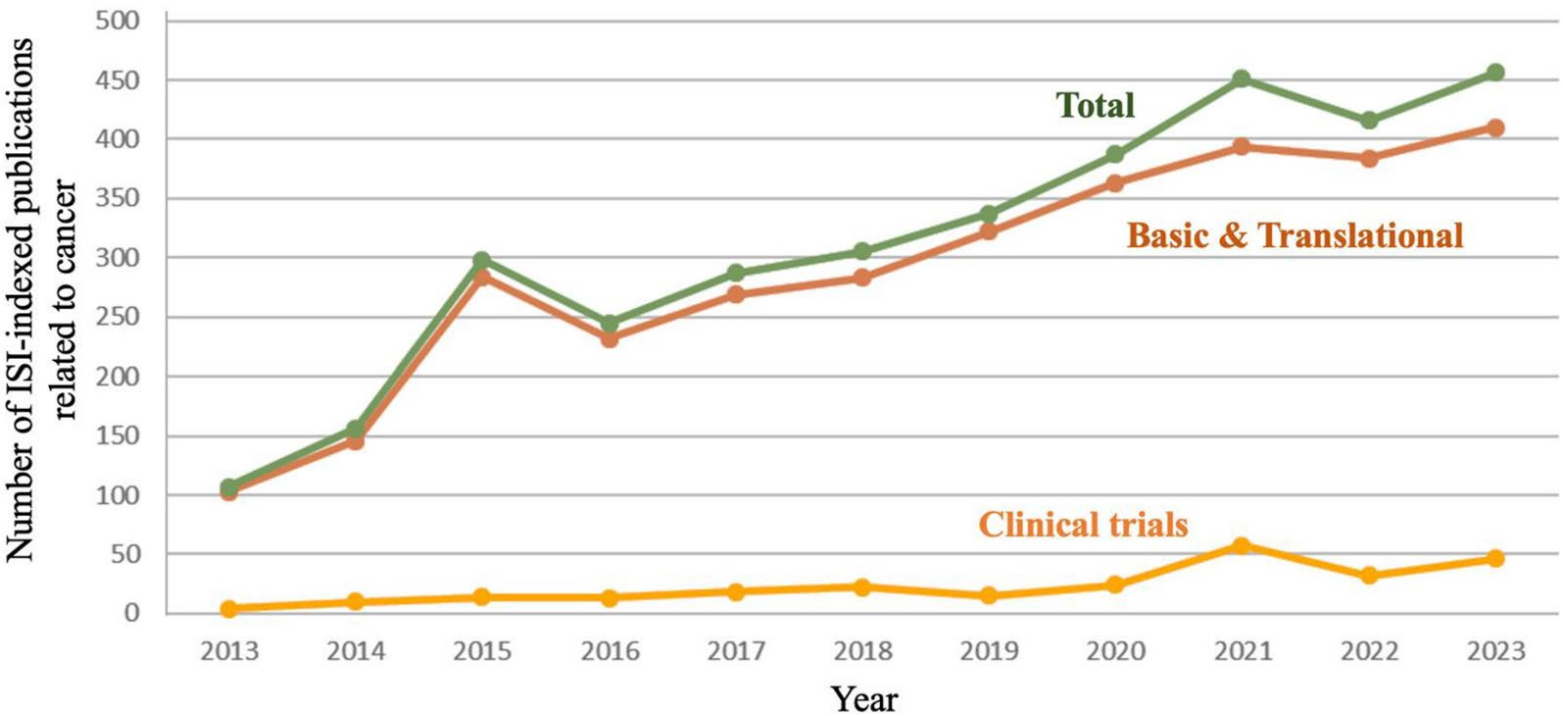
Cancer-related NCBI-PubMed indexed publications in Chile 2013–2023. All cancer-related papers are considered basic and translation except for papers reporting results from clinical trials

As can be seen in Fig. 10, most papers published in Chile are basic science or translation research. The majority (66%) of the publications come from traditional universities and 27% are from hospitals or clinics. However, this may not be a true reflection, as the two principal universities possess affiliated teaching hospitals, making it difficult to classify a paper originating from the university or the hospital.

Of the 350 academic oncology-related papers in 2023, roughly 200 are published by either the Universidad de Chile or the Pontificia Universidad Católica de Chile. The only other universities to publish more than 20 cancer research papers in any given year are the Universidad de la Frontera, the Universidad de Desarrollo and the Universidad de Concepción (please refer to Supplementary Fig. 1). As most of the laboratories in Chile were closed for a year due to the COVID-19 pandemic, many universities had their highest number of publications in the year 2021 before a dip in 2022 and recuperation in 2023.

In a previous study, between 2014 and 2019, it was observed that Chile published twice the amount expected from the Latin American trend-line, although the overall rate was below European levels [94]. As reported previously [21] the flow of academic publications in cancer has been increasing modestly but steadily over the last three decades. Maybe surprising, there is still an increase in publications despite a plateau in funding in the same period. This demonstrates the versatility of researchers; however, the metric of number of papers does not necessarily reflect quality. A crude calculation dividing the number of basic/translation science publications by the total investment in this area yields a value of approximately three papers per US$100,000 investment, which is in line with reports from high income countries [95].

Only a more detailed analysis, which classifies publication by investigation, case reports, and impact factors and/or citations, will give a true reflection of how the plateau in national funding is affecting scientific productivity and explain why there is an increase in productivity despite a plateau in funding. However, to address this question we performed an analysis of the number of review articles between the years 2015 and 2023. We omitted the year 2019 due to many of the laboratories in Chile having to close for safety reasons during a period of civil unrest in the country in the latter end of that year. Furthermore, following this civil unrest Chile experienced one of the strictest quarantines in Latin America with many laboratories being closed for between one to two years. In Fig. 10 the peak observed in publications in 2021 was due to 109 review manuscripts (24% total publications) in cancer being published that year, which fell back to 69 (16.5%) in 2022. Except for 2021, the percentage of review articles in cancer in Chile has remained steady at 15–18% since 2016.

In Fig. 10 we also observe an increase in clinical trial papers in the last few years which reflects the increasing number of clinical trials in the country (compare Fig. 8). However, it is interesting that a clinical trials budget of US$100 million produces six-fold less publications than US$10 million of basic research funding. While the clinical trials started in 2019 may still be ongoing and/or in data analysis, this is in line with global observations that there are often scarce scientific and clinical results disseminated after a clinical trial.

### Gender equality in cancer research in Chile

**Fig. 11:**
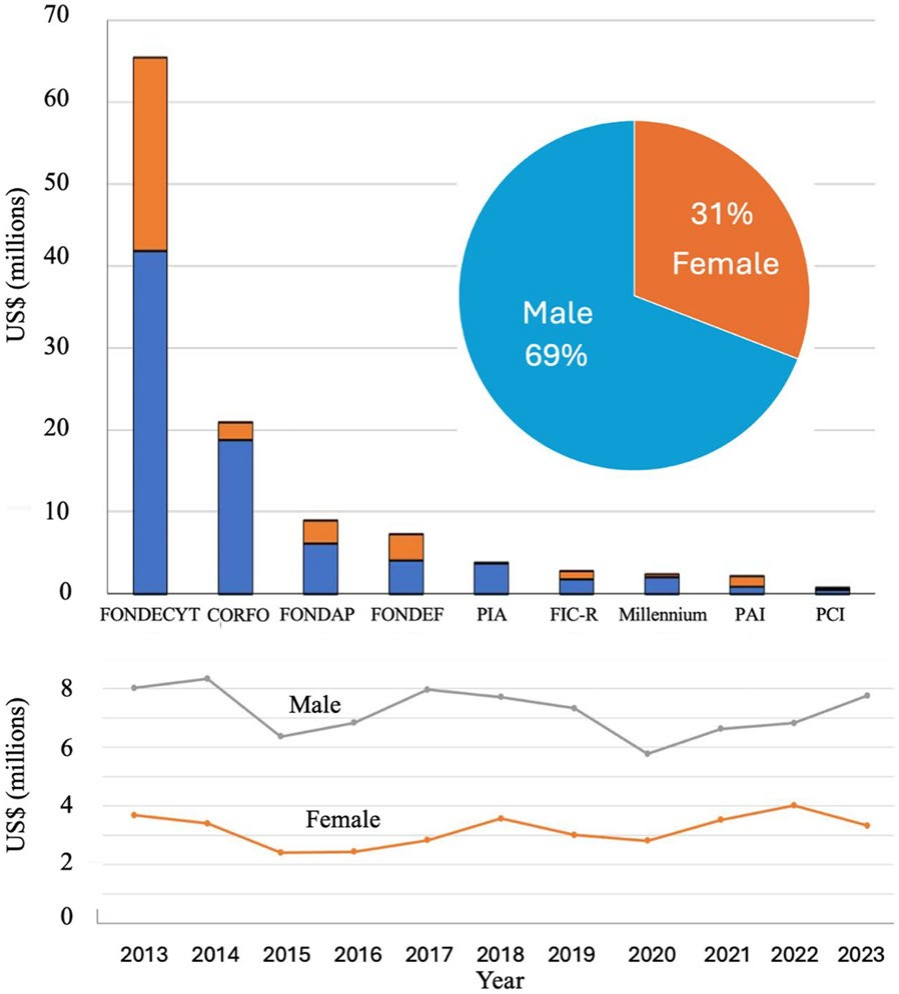
Gender equality in cancer research-related investigation in Chile. Only national non- clinical trial projects are considered. A. Pie graph represents the total number of projects awarded between 2013–2023. The bars represent distribution by sex within the individual national funding source. B. Line graph represents the total money awarded within nationally funded research grants by sex between 2013–2023

Gender equality in Chile is now discussed in all sectors of society. Chile possesses the most favorable indicators in gender equality (measured by the Gender Inequality Index) in Latin America and the Caribbean, despite this, there are still substantial improvements to be made [96]. One of the first initiatives of the newly formed Ministry of Science, Technology and Knowledge was to conduct a study into gender discrimination in the awarding of research grants. This approximation of gender was performed using only the biological sex assigned at birth (male or female) of the investigator, which is the only current available information on ministerial databases. The study found that specifically for first time applicants, women were more likely to receive a lower score on their grant proposals. Furthermore, women in science were more likely to abandon their academic career than male counterparts. A difference in salary was also detected in professionals, and this was greater for the STEM (Science, Technology, Engineering and Mathematics) areas [97]. There exists literature reporting gender gaps in Chilean science although the social mechanisms underlying this phenomenon remain to be fully understood [98–100]. These publications have suggested that homophily of gender is evident and practiced by men but not necessarily by women. In other words, a male scientist regards another male as a peer or reference, while a female scientist also regards a male scientist among her peers. The authors speculate that the reproduction of stereotypes is expressed in scientific orientation to the male gender, thus favoring male recruitment and selection [100]. Thus, attempts by woman to pursue a scientific career is hindered by a male domination stereotype, which may have been seeded in primary school when interests in science are built. A previous publication from Chile which reported a dominance of males in the technology sector, a dominance of females in the health sector and a general 50/50 division within the sciences in the year 2017 [99]. The universities are also monitoring gender distribution and equality. Taking as an example the two principal universities in Chile responsible for scientific research, the University of Chile and the Pontificia Universidad Católica de Chile, in general no differences were observed in enrolled of undergraduate students classifying themselves as male or female in the scientific disciplines. In line with a previous report by Dinamarca and colleagues in 2020 [98], reporting that the gender gap in Chile increases as the academic career progresses, an *in house* study conducted by the University of Chile in 2021 demonstrated that in the scientific disciplines, 54% of the 6,109 students were female, however, this percentage dropped to 44% in the postgraduate program and to an average of 28% in members of academia obtaining funding [101]. Mirroring the University Chile, 62% of the current academics (in all disciplines) at the Pontificia Universidad Católica de Chile are male. In the area of health, 54% of academics in this university are male, however, this proportion is higher in other STEM areas in the university [102]. At the national levels, 79% and 64% of the undergraduates in STEM related disciplines are male at the pregraduate and doctoral levels respectively [100]. As observed in Fig. 11, an analysis of national public funded cancer-related projects gives a similar pattern, with 69% of the projects being led by men and this has been constant over the last decade. These proportions in cancer funding most likely reflect the male bias in STEM-related academia and thus percentage of men applying for research grants. Both the Ministry and the universities are proposing actions to correct this inequality.

## Conclusions and commentaries

Chile has recognized the burden of cancer and has implemented a Cancer Law and a National Cancer Policy to address the lack of national infrastructure and human resources, however there is still a long road to achieve median OECD statistics and provide greater equality in access to treatment in the public health system. Chile's current younger generation consumes less tobacco and alcohol than before, and therefore (potentially), a Ministry led attack on national obesity could make serious inroads into reducing the future incidence of cancer in the population.

In 2015 we presented a table of 11 key steps to improve oncology care and cancer research. Of these 11, three have been completed in their entirety, six are in various degrees of progress, and two have not been implemented. As these steps are just as relevant today as a decade ago, below we provide an update on each step and discuss further directions in which their implementation may be assisted.

***Step 1. “Publicly recognize the imminent burden of cancer and implement a strong and visible National Cancer Policy”, and Step 2. “Introduce a national cancer law”.***

Each step has been implemented by the formation of the National Cancer Plan in 2018 and the establishment of a cancer law in 2020 that guarantees the appraisal of the cancer care every 5 years. However, this objective does not stop here, it is the implementation of this law and the maintenance of financing that is now the priority.

***Step 3. Create integrated cancer hospitals. The National Cancer Institute (Instituto Nacional de Cáncer) in Santiago would be the prime candidate to transform into an integrated cancer hospital.***

Currently the concept of specialized cancer hospitals is under discussion in the Ministry of Health. Chile has recognized the need to create centers which specialize in specific cancers (e.g. digestive tract cancer surgeries) and is now implementing a logistic analysis to determine the best location for these centers in the country. Over the last 90 years the Chilean National Cancer Institute (INC) has played a fundamental role in the resolution of oncological pathology in the country. While technically a hospital more than an Institute, the building suffered serious damage during the 2010 earthquake. Plans to build a new INC have been announced, where along with the new construction, the number of available beds will increase from 81 to 249, the number of complex operating theaters from 3 to 13 and the number of annual consultations from a current 46,307 to 251,605 [103]. Chile also possesses a private clinic/non-governmental organization practicing uniquely in oncology. La Fundación Arturo López Pérez (FALP) is one of the main integral cancer care centers and has invested heavily in state-of-the-art technology over the last decade. FALP is now planning to open new centers in the northern and central Chile to meet the growing demand in oncology. New hospitals and centers specializing in specific cancer types are currently under consideration in the Ministry of Health. These measures, together with the strengthening of the cancer diagnosis and treatment capacity in the health network, would allow expanding the guarantees and health problems included in the GES policy. Furthermore, generating both incentives in incorporating clinical research and the stimulation of patient recruitment will increase the access of patients in the public sector to clinical trials.

***Step 4. Conduct regular systemic nationwide studies on the status of oncology at all levels. Perform regular evaluations of the impact of health and cancer reforms. Promote studies of the economics burden of cancer to inform a National Cancer Policy.***

A national cancer plan is now in place for 2018–2025 (updated 2022–2027) and National Cancer Commission has been established. A department of cancer has been formed within the Ministry of Health and the permanent dedicated staff increased from 3 to 60 people. In 2022 the Ministry of Science, Technology, Knowledge and Innovation awarded a FONDAP (CECAN) to the Pontificia Universidad Catholic de Chile and the Universidad de Chile (with participation with numerous other academic centers). This Center has brought together experts in public health, medicine, scientific investigation, and epidemiology to gather evidence to aid ministerial policy making decisions.

***Step 5. Initiate nationwide prevention programs incorporating all sectors of society.***

While nationwide prevention programs in the form of a new tobacco law, a tax on sugar beverages and the incorporation of etiquettes on all foodstuffs are ongoing (discussed above), this step is probably never ending, and it is necessary to strengthen and innovate in new approaches that consider a health determinant. As in many parts of the world the public knowledge and understanding of cancer in Chile is poor. Mythology still exists around its causes and the word is still considered a death sentence. Public education in oncology now falls to the universities and the investigators to demystify cancer and disseminate the message of cancer risk and screening, thus contributing to policies by fostering genuine, impacted and empowered social participation and inclusion of strategic actors, for the generation of healthy living environments.

***Step 6. Promote the investigation in cancers of high national relevance. (e.g. stomach, gallbladder and arsenic-related lung cancer in Chile). We suggest the setting up of specific task force. Lifting any import tax on reagents and literature destined for scientific research and education.***

The historic presence of arsenic in the drinking water in the Northern part of Chile remains a factor for cancer incidence in this region. However, arsenic levels in the water supply are now tightly monitored and controlled. As mentioned, both stomach and gallbladder cancer, whose high incidence are related with underdeveloped countries, have been lowered. As discussed previously, the incidence of gallbladder cancer has reduced drastically, however after an initial period of decline, stomach cancer rates have now stabilized and remain higher than expected. Thus, this original suggestion for a stomach cancer task force may remain relevant and studies to test and potentially implement the hypothesis that *H. pylori* eradiation will further reduce cancer incidence will require state financing. Further studies specific to the Chilean population are examining if early diagnosis markers (for example pepsinogen levels and specific *Helicobacter* genotypes) can help with screening and reduce the long national waiting list for endoscopies.

The lifting of any import tax on reagents and literature destined to scientific research has not been openly discussed and appears to be an unlikely option for Chile at this time.

***Step 7. Partner with other South American countries to pool resources on regional problems.***

Through coordination from King’s College London and Guy’s Hospital London, Latin American organizations representing the region, have collaborated on a publication to determine metrics within cancer research in the region [94]. The clinical trials group GOCCHI also extended conversations with their counterparts in other South American countries and with North American (notably the SouthWestern Oncology Group (SWOG)) and European organizations. Through funding received from the European Union’s Horizon 2020 research and innovation program, *EquityCancer-LA* has continued to build on previous work 2009–2013 and 2013–2019 to analyze access to care and the effectiveness of the implementation of a multicomponent integrated care intervention to improve early diagnosis of cancer in the public health service networks of Chile and its regional neighbors Colombia and Ecuador [104]. However, the concept of ministries uniting to tackle the burden of cancer or the promotion of clinical trials in the region, remains a challenge for the coming decade.

***Step 8. Specifically focus on clinical trial promotion to stimulate pharmaceutical company interest in Chile and clinical oncologist participation in research.***

Chile could be established as a hub for Clinical Trials in South America. With a stable economy and greater transparency that most Latin American countries, the country could become a springboard for pharmaceutical companies (among other sponsors). New legislation and incentives to encourage public hospital participation in clinical trials could help in the attraction. The Ministry of Health must implement revisions in the national normative for clinical investigation and the corresponding ethical framework. In accordance with the spirit of the new Cancer Law, legislation is needed to guarantee a workable legal framework for both the promotion and implementation of clinical trials and coordinate the analysis and potential modifications of this legislation. Please refer to the ethical committee requirements below.

***Step 9. Promote incentives, particularly financial, to further stimulate the private sector investment, coupled with stimulation of capital risk and angel investments in the biomedical field.***

In the United Kingdom, the charity Cancer Research UK spent £415 (US$500) million in the 2022/2023 financial year. The approximately 620 different UK organizations in the Cancer charity sector funded between them 60% of all national cancer research [105]. Taking this example, the Chilean government should prioritize the consideration of changes in the law of donations and offer incentives to develop a plan of attraction for public and private capital in oncological research. Charities and voluntary agencies are not limited to only raise money for research, but also to improve the hospital and patient support infrastructure together with patient and public education, hospice and advice. However, this will require non-profit institutions to organize better and present projects to generate social impact. Furthermore, these incentives should bring into the Chile the international private sector to establish research centers and contact more clinical trials.

***Step 10. Create, strengthen and expand of regional cancer registries.***

The recent enactment of the cancer law and the national cancer plan, which includes strengthening registration, information, and surveillance systems as its strategic goal number 4, mandates the existence and functioning of a national cancer registry with mandatory reporting. The objective of this registry is to provide relevant information about the disease to support scientific research and contribute data, along with other information sources, for cancer epidemiological surveillance, thereby facilitating decision-making in the management of cancer patient care.

There has been extensive debate over whether this registry should be based on information from public and private pathology services or institutional cancer registries. The absence of fully functioning hospital cancer registries in all health institutions, coupled with the usual systematization and digitalization present in pathology laboratories (both public and private), makes this source of information more feasible. The Ministry of Health has defined in its resolution N°173 of January 31, 2024, that the national cancer registry corresponds to the amalgamation of information sources generated in the national cancer registry platform, the national pediatric cancer registry, hospital cancer registries, population-based cancer registries, and any other sources related to the disease.

The implementation of this registry's operation should occur during the year 2024, and only upon completion of this implementation will we be able to evaluate whether the implementation strategy and the data obtained from this registry will be useful for public health decision-making regarding cancer in Chile. However, it is undeniable that the centralization of digital information will enable patient traceability throughout the entire care process, from detection to treatment delivery, referrals, and subsequent follow-ups.

***Step 11. Promote the formation of medical cancer specialists (e.g. oncologists, palliative care, nursing and other cancer-related human resources).***

While the field of cancer treatment is usually broken down into the areas of medical, radiation, and surgical oncology, numerous other disciplines such as palliative care, psychiatry, nursing, kinesiology, pharmacy, nutrition among many others require specialist training to provide optimal patient care. Trained specialists in oncology are lacking in every sector of cancer care throughout the country. Although an exact figure is unknown, there are approximately 160 oncologists in Chile, of which 93 have some participation in the public system [106]. However, to be in line with other developed countries of the OECD, these values still leave Chile well below the half the number of oncologists per million inhabitants [107]. While not fulfilling the national requirement to provide equitable patient care in oncology to lack of medical specialists, a further problem is that 53% of the oncologists in the country’s public network are concentrated in the Metropolitan Region, which produces both physical and economic inconveniences to patients who need to travel to the capital from the Regions [106]. The National Cancer Law enacted during 2020 establishes the requirement for training human resources for cancer treatment, considering medical specialists, health professionals, and researchers in the field. A challenge the Ministry of Health and the universities in Chile will be to increase the oncology workforce in all areas in the coming years.

## New key steps to improve cancer care and research

Together with the 11 previously mentioned steps, we have identified three further measures that will aid public health in the oncology sector in Chile.

### Optimize Research Ethics Committee performance, biobanks and the legal framework for promotion and implementation of clinical trials

The Research Ethics Committees (RECs) have the purpose of carrying out the ethical review of research protocols involving human subjects, thus protecting the rights, safety and well-being of participants in research projects. In Chile, these committees are constituted as collegiate and independent organizations housed in public or private institutions. In the last decade, the number of RECs has increased from 36 to 60, with 70% located in the Metropolitan Region. Despite significant progress, gaps are still recognizable, and challenges remain for better performance.

Regarding RECs accreditation, this process tends to focus on the formal aspects of its operative process, in accordance with compliance to the regulations, without delving into the analysis of the criteria, deliberation processes and internal decision-making, which lead to the approval or rejection of a specific protocol.

The authors feel that a national registry of clinical studies would be desirable. The implementation of a national registry should be the responsibility of the health authority, with the aim of making the information on the evaluated research protocols transparent and facilitating communication between different RECs. Regarding current RECs operation, there are problems related to the harmonization of review criteria between the different committees, as no formal mechanisms have been established that allow standardization of the criteria required to carry out the revision of multicenter research protocols.

The growing complexity of oncological studies requires improving the skills and training of RECs members, incorporating trained human resources in oncology and innovative research methodologies. Likewise, the development of biobank networks within the country will also require an improvement in the REC capabilities, as members with specific expertise will be needed as future members of biobank advisory committees.

Finally, new legal frameworks, such as the Ricarte Soto law (Law No. 20,850) and the Cancer law (Law No. 21,258) and their interpretation have generated an intense debate and made strenuous work for the RECs. In particular, an area that needs to be further defined is the protection for participants in clinical trials after finalization of the study, if the treatment needs to be continued afterwards. Another area of debate and potential reconsideration is the role of the health facility director in the authorization of patient recruitment for extramural clinical trials.

### Preparation in Chile for the era of precision medicine

Genomics can identify individuals at higher risk of developing certain cancers based on their genetic profile, enabling more efficient preventive measures and early screening and tumor genomic profiling can help determine the likelihood of response to therapy. While the National Cancer Law recognizes the right to genetic counseling, obstacles that must be overcome are the lack of coverage for genetic testing, insufficient infrastructure in the public system, and the shortage of professionals trained to provide genetic counseling. Private laboratories are offering testing options and public institutions have started to acquire Next Genome Sequencing (NGS) capabilities; however, implementation is incomplete. In public health, Hospital Salvador in the capital Santiago currently offers NGS analysis for hematological neoplasia, while the ISP has received the technology transfer of NGS assay “TumorSecTM” from investigators at the Universidad de Chile that is currently in the process of validation and approval [108, 109, 110]. It is crucial for authorities, healthcare professionals, and civil society to lobby for genetic testing financial coverage within the national and private health system and this could accelerate the development and implementation of diagnostic laboratories. Additionally, characterizing germline and somatic variants in the Chilean population is crucial for understanding cancer epidemiology and developing tailored treatments.

### Treatments of tomorrow and national current Good Manufacturing Practice (cGMP) manufacturing in biomedicine

A strategy for accelerating patient access to advanced therapies and the implementation of Chimeric antigen receptor T-cell (CAR-T) and other gene therapy technologies in Chile is required. Despite being innovative and extremely promising treatments for patients with previously treated hematologic malignancies, currently, only Brazil in the South and Central American region has active ongoing CAR-T clinical trials. The hurdles are many, including compliant manufacturing facilities, technical guidelines, clear and applicable regulations covering everything from quality control to clinical trials and regulatory approval. As a result, these therapies face a long journey to reach national patients. The cell and gene therapy industry maybe confronted with an insurmountable barrier, as Chile lacks the minimum conceptual or operational regulatory framework. In 2024 the Chilean ISP published the first Technical Guide for Biological Therapies, based on WHO guidelines; although before implementation, this plan must negotiate a complex legal scenario. However, there are current strategies and initiatives to implement this legislation and technology in Chile. State funds have sponsored matching funding for 10 years for the creation of the center of excellence IMPACT (located at the Universidad de Los Andes, Santiago). The mission of this Center is to make cell and gene therapy accessible to the Chilean population. Furthermore, CORFO have awarded the same investigators a six-year fund to establish a certified cGMP manufacturing center will be referred to as “R.MATIS”. The ISP will participate in the facility’s technical and strategic boards. Public engagement and health gremials advocating for innovation for the benefit of patients is still required, as is establishing the groundwork for a regulatory framework, technology transfer agreements and participation in future clinical trials [111].

## Data Availability

All data represented is this manuscript was obtained from publicly available databases. Any analysis, datasheets or tables generated in the preparation of this article will be made publicly available upon written request to the corresponding author.
